# Plant Flavone Apigenin Binds to Nucleic Acid Bases and Reduces Oxidative DNA Damage in Prostate Epithelial Cells

**DOI:** 10.1371/journal.pone.0091588

**Published:** 2014-03-10

**Authors:** Haripaul Sharma, Rajnee Kanwal, Natarajan Bhaskaran, Sanjay Gupta

**Affiliations:** 1 Department of Urology, Case Western Reserve University, Cleveland, Ohio, United States of America; 2 Department of Urology, The Urology Institute, University Hospitals Case Medical Center, Cleveland, Ohio, United States of America; 3 Department of Nutrition, Case Western Reserve University, Cleveland, Ohio, United States of America; 4 Division of General Medical Sciences, Case Comprehensive Cancer Center, Cleveland, Ohio, United States of America; Jawaharlal Nehru University, India

## Abstract

Oxidative stress has been linked to prostate carcinogenesis as human prostate tissue is vulnerable to oxidative DNA damage. Apigenin, a dietary plant flavone, possesses anti-proliferative and anticancer effects; however, its antioxidant properties have not been fully elucidated. We investigated sub-cellular distribution of apigenin, it’s binding to DNA and protective effects against H_2_O_2_-induced DNA damage using transformed human prostate epithelial RWPE-1 cells and prostate cancer LNCaP, PC-3 and DU145 cells. Exposure of cells to apigenin exhibited higher accumulation in RWPE-1 and LNCaP cells, compared to PC-3 and DU145 cells. The kinetics of apigenin uptake in LNCaP cells was estimated with a K_m_ value of 5 µmole/L and V_max_ of 190 pmoles/million cells/h. Sub-cellular fractionation demonstrated that nuclear matrix retains the highest concentration of apigenin (45.3%), followed by cytosol (23.9%), nuclear membranes (17.9%) and microsomes (12.9%), respectively. Spectroscopic analysis of apigenin with calf-thymus DNA exhibited intercalation as the dominant binding mode to DNA duplex. Apigenin exposure resulted in significant genoprotective effects in H_2_O_2_-stressed RWPE-1 cells by reduction in reactive oxygen species levels. In addition, apigenin exposure suppressed the formation of 8-hydroxy-2′ deoxyguanosine and protected exposed cells from apoptosis. Our studies demonstrate that apigenin is readily taken up by normal prostatic epithelial cells and prostate cancer cells, and is incorporated into their nuclei, where its intercalation with nucleic acid bases may account for its antioxidant and chemopreventive activities.

## Introduction

Prostate cancer has the highest incidence of any cancer in American men and is the second leading cause of cancer-related mortality [1]. The American Cancer Society estimates that in 2013, approximately, 238,590 new cases of prostate cancer were diagnosed and 29,720 men died of this disease [1]. Although the reasons for this high incidence are unknown, human prostate tissue may be particularly vulnerable to oxidative DNA damage by free radicals which are thought to play a critical role in the multi-step process of carcinogenesis [2]–[4]. Several etiological factors have been proposed in the genesis of prostate cancer, including increased cellular turnover, loss of DNA repair enzymes, impairment of antioxidant signaling network and persistent chronic inflammation in the prostate gland [5]–[9]. The resulting oxidative stress, characterized by the generation of reactive oxygen and nitrogen species in the local milieu, produces permanent genomic alterations and cellular DNA damage marked by accumulation of 8-hydroxy-2′ deoxyguanosine (8-OHdG). Studies demonstrate that 8-OHdG is the most prevalent DNA damage product and when incorporated into DNA leads to point mutation via an A→T substitution [3], [10]. We have previously demonstrated that persistent chronic inflammation in the prostate gland, associated with increased accumulation of 8-OHdG in prostatic epithelium, promotes premalignant and malignant changes [9], [11]. Conversely, reduced 8-OHdG levels, consistent with reduced oxidative stress, have been reported in subjects receiving plant-based diets rich in flavonoids and polyphenols [12]–[15]. These diets are characterized by conspicuous consumption of green tea and plant flavones rich in apigenin.

Apigenin (4,5,7-trihydroxyflavone), a flavone subclass of flavonoid widely distributed in many herbs, fruits, and vegetables is a substantial component of the human diet and has been shown to possess a variety of biological characteristics, including chemopreventive activity and tumor growth inhibition [16]. Recent studies in several biological systems have shown that apigenin possesses anti-proliferative properties, and induces cell cycle arrest and apoptosis in various human and animal-derived cancer cell lines [17]–[20]. In transformed mouse liver cells, apigenin has been reported to reduce the toxicological effects of dioxin by suppressing the dioxin-induced activation of the aryl hydrocarbon receptor [21]. After dietary intake, apigenin becomes widely distributed in various tissues and is known to exert beneficial effects [22]. Apigenin has been shown to protect endothelium-dependent relaxation of rat aorta against oxidative stress [23]. Furthermore, apigenin intake results in reduced levels of lipid peroxidation products and increased antioxidant enzymes, preventing hepatocarcinogenesis in rats exposed to N-nitrosodiethylamine and phenobarbitol [24]. In addition, the bioavailability of apigenin has also been investigated in animals and human subjects. Short-term intake of apigenin-rich parsley by healthy human subjects increased the level of antioxidant enzymes erythrocyte glutathione reductase and superoxide dismutase [25]. However, the cellular distribution of ingested apigenin, its uptake in sub-cellular compartment and its anti-oxidative activity has not been fully elucidated.

In this study, we determined the sub-cellular distribution of apigenin in prostate cancer and normal prostate epithelial cells. We also studied the protective role of apigenin against oxidative stress caused by hydrogen peroxide. Our results demonstrate that apigenin preferentially accumulates in the nuclear matrix, particularly binds to nucleic acid bases and has the ability to reduce oxidative DNA damage in prostate epithelial cells.

## Materials and Methods

### Chemicals and Reagents

All chemicals and reagents were purchased from Sigma Chemical Co. (St. Louis, MO) unless otherwise specified. Tissue culture supplies were procured from Falcon (Becton-Dickinson Labware, Franklin Lakes, NJ). All tissue culture reagents and 2′, 7′-dichlorofluorescein diacetate (DCF-DA) was purchased from Invitrogen (Grand Island, NY) whereas fetal bovine serum was purchased from Tissue Culture Biologicals (Tulare, CA).

### Cell Culture

Human prostate cancer LNCaP, PC-3 and DU145 cells and transformed human prostate epithelial RWPE-1 cells were obtained from American Type Culture Collection (Manassas, VA). LNCaP, PC-3 and DU145 cells were cultured in RPMI 1640 medium containing 10% fetal bovine serum supplemented with 1% penicillin-streptomycin at 37°C with 5% CO_2_. RWPE-1 cells were cultured in keratinocyte growth medium supplemented with 5 ng/ml human recombinant epidermal growth factor and 0.05 mg/ml bovine pituitary extract (Invitrogen, Carlsbad, CA).

### Apigenin Stability

To measure the stability of apigenin during incubation was performed by incubating PC-3 cells with 20 µM apigenin in RPMI 1640 medium containing 10% FBS, and same concentration of apigenin incubated in culture medium without cells under similar culture conditions. Apigenin levels in the medium were detected at set intervals using HPLC.

### Cellular Uptake of Apigenin

Human prostate cancer LNCaP, PC-3 and DU145 cells and human prostate epithelial RWPE-1 cells were seeded at a density of 1×10^5^/mL in 100 mm culture plates with three replicates for each incubation time point. After 24 h of seeding the culture medium was replaced with fresh medium containing 20 µM apigenin. The time course of apigenin uptake by each cell line was determined by incubation with medium containing 20 µM apigenin for up to 16 h.

### Kinetics of Apigenin Uptake

Because of higher uptake of apigenin by LNCaP cells, these cells were further studied for uptake absorption kinetics and sub-cellular distribution of this compound. Cells were grown as triplicate cultures in RPMI 1640 medium supplemented with 10% fetal bovine serum and at various concentration of apigenin range from 1.25 µM to 20 µM up to 6 h. At each time point, cells were counted using a hemocytometer, and cellular apigenin was extracted and then measured using HPLC as described previously [26]. Finally, the kinetics of apigenin uptake by LNCaP cells was evaluated using the Michaelis-Menten kinetics model.

### Sub-cellular Distribution of Apigenin

LNCaP cells were cultured in triplicates in RPMI 1640 medium supplemented with 10% FBS and apigenin at final concentration of 20 µM. After 48 h of incubation, cells were harvested and washed with cold PBS. Cells were separated by centrifugation at 600×g for 10 min at 4°C. Sub-cellular fractionation was carried out as previously described with some modifications [26]. Cell pellet were resuspended in hypotonic buffer containing 20 mM Tris-HCI, pH 7.4, 5 mM MgCl_2_, 5 mM CaCl_2_, 1 mM DTT 1 mM EDTA and protease inhibitor cocktail for 45 min. Cytosolic fraction having microsomes was separated by centrifugation at 2000×g for 30 min at 4°C. The supernatant was removed and then ultra-centrifuged for 3 h at 100,000×g at 4°C to separate the microsomal fraction. The crude nuclear pellet from the low-speed centrifugation was resuspended in ice-cold low salt buffer containing 20 mM Tris-HCl, 5 mM MgCl_2_, 2 mM KCl, 1 mM DTT, and 1 mM EDTA with protease inhibitors for 30 min. Then high salt concentration buffer containing 20 mM Tris-HCI, pH 7.4, 5 mM MgCl_2_, 1.2 M KCl, 1 mM DTT, 1 mM EDTA and protease inhibitors was added drop wise at 4°C with constant stirring for 30 min and centrifuged for 30 min at 25000×g at 4°C to separate the nuclear matrix from nuclear membranes. All four fractions were concentrated to dryness by vacuum evaporation and reconstituted in 50% ethanol prepared in PBS to deproteinated the sample. After centrifugation at 10000×g for 10 min, supernatants were subjected to HPLC analysis to analyze apigenin content.

### Apigenin Binding with DNA

Calf thymus (CT) DNA was prepared in double distilled water adjusted to pH 7.2, sonicated and filtered through a 0.45 µM filter. It was kept stirring for overnight at 4°C to obtain a homogeneous solution of polymerized DNA. Aqueous solution of apigenin was freshly prepared. Experiments were performed in 0.1 M phosphate buffer solution, with pH 7.4. 0.25 µM DNA solutions were prepared having varying concentrations of apigenin ranging from 0.06 mM to 0.4 mM. Absorption spectra of all solution were recorded from 230 nm to 500 nm using NanoDrop 1000.

### Spectrum of Calf Thymus DNA Treated with Hydrogen Peroxide and Apigenin

CT-DNA was incubated with hydrogen peroxide (H_2_O_2_) at physiological pH 7.4. The reaction mixture consisted of 0.25 mM CT-DNA, and various concentration of H_2_O_2_. In another reaction 0.25 mM CT-DNA, mix with various concentration of apigenin then treated with H_2_O_2_. The incubation was carried out for 2 h at 37°C. Spectra were recorded with UV-visible spectrophotometer.

### Reactive Oxygen Species Measurement

RWPE-1 cells were plated at 1×10^5^ cells per well in 96-well plates in appropriate culture medium. As cells reached to 75–80% confluence subsequently treated with different concentration of apigenin for 16 h and 200 µM H_2_O_2_ for 6 h. The treatment medium was removed and cells were washed with phosphate-buffered saline (PBS) and than exposed to PBS containing 10 µM 2′, 7′-dichlorofluorescein diacetate (DCF-DA), a dye that fluoresces when ROS are generated. The cells were incubated with DCF-DA for 20 min, after which fluorescence intensity was determined using FluoStar Omega Spectrophotometer (BMG Labtech) at 480 nm excitation and 560 nm emission as previously described [27]. The values, expressed in percentage arbitrary fluorescence units, were compared across treatment groups.

### 8-OHdG Measurement

Measurement of 8-OHdG in cultured cells was performed with OxiSelect Oxidative DNA damage ELISA kit, Cell Biolabs, Inc. (San Diego, CA) as per vendor’s protocol. Briefly, DNA was converted to single stranded DNA and 8-OHdG was quantified by quantitative ELISA assay. The quantity of 8-OHdG in the specimens were determined by comparing its absorbance with known 8-OHdG standard curve as previously described [11].

### Flow Cytometry–annexin V Assay

To distinguish the proportion of viable cells from cells undergoing apoptotic death, propidium iodide (PI) and annexin V staining assays were employed. RWPE-1 cells were treated with 10 µM and 20 µM of apigenin for 16 h alone or further incubated for 6 h with 200 µM H_2_O_2_. Later, the cells were harvested, washed twice with PBS, stained with PI and annexin V and analyzed using FACS cytometer as described previously [27].

### Statistical Analysis

The experiments on cell culture and CT-DNA were repeated at least three times. Results were expressed as mean values ± SD. Statistical comparisons were made by ANOVA followed by a Dunnett’s multiple comparison test. *p* values <0.05 were considered significant.

## Results

### Cellular Uptake and Stability of Apigenin

**Figure 1:**
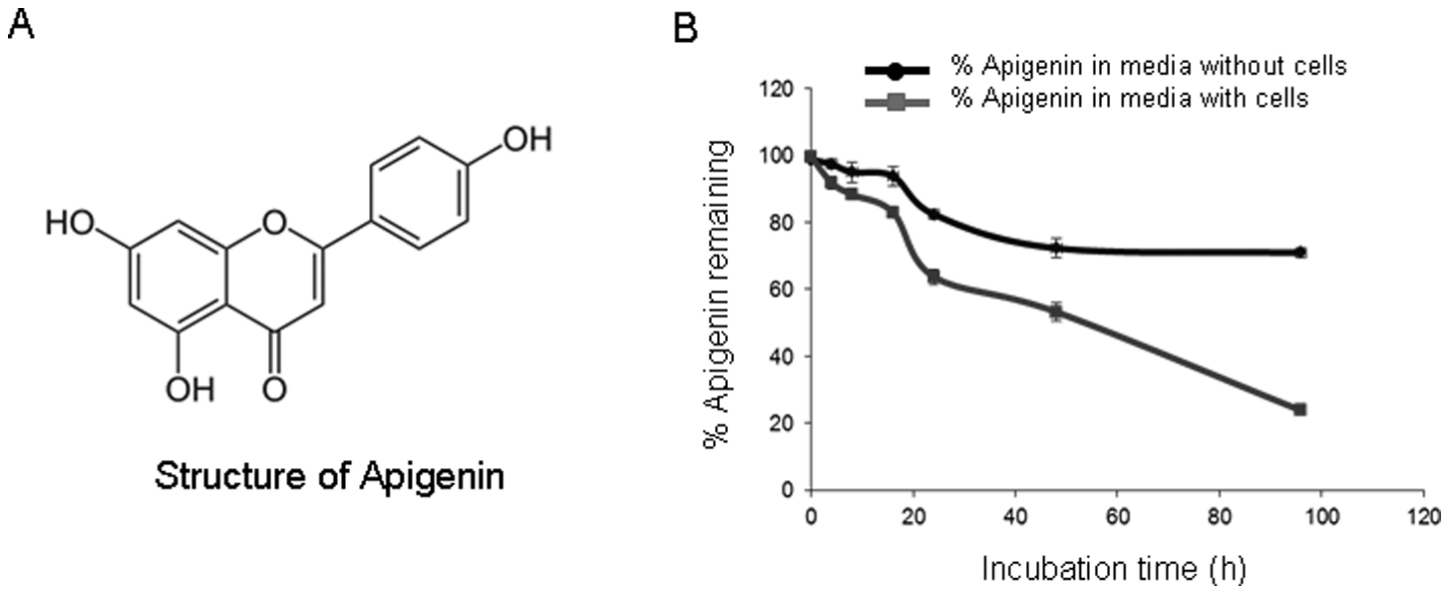
Chemical structure and stability of apigenin. (**A**) 4′, 5, 7-trihydroxyflavone (**B**) Apigenin stability was determined at 37°C by incubating 20 µM apigenin with or without human prostate cancer PC-3 cells for up to 96 h. The concentration of apigenin at each time point was measured using UV-HPLC. Points±SD, percentage of remaining apigenin performed three times. Details are described in materials and methods section.

The structure of apigenin is provided in **figure 1A**. To measure the stability of apigenin during the incubations, apigenin was dissolved in dimethyl sulfoxide, added in the culture medium to attain 20 µM concentrations and incubated with or without PC-3 cells for up to 96 h. More than 70% of apigenin remained in cell culture medium without PC-3 cells after 96 h whereas 30% loss of apigenin may be due to degradation. The concentration of apigenin in the medium of incubation containing PC-3 cells was lower than that of the corresponding incubation without cells which decreased to 76% at 96 h post-incubation **(** **Figure 1B** **)**. This difference was probably due to cellular uptake of apigenin. Overall, these studies demonstrate that there is a significant uptake of apigenin by the cells.

### Comparison of Apigenin Uptake by Non-tumorigenic and Prostate Cancer Cells

**Figure 2:**
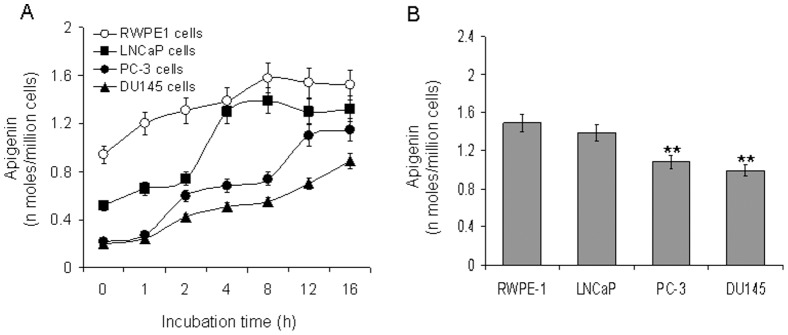
Uptake of apigenin by various human prostate cell lines. (**A**) Time course apigenin uptake by transformed human prostate epithelial RWPE-1 cells and prostate cancer LNCaP, PC-3 and DU145 cells incubated with 20 µM apigenin for up to 16 h. Cellular uptake of apigenin (apigenin/million cells) was determined using UV-HPLC. (**B**) Bar graph of apignin uptake by various cell lines at 16 h. Bars±SD of experiments performed three times. **P<0.001. Details are described in materials and methods section.

In the next experiment, LNCaP, PC-3 and DU145 prostate cancer cells as well as transformed human prostate epithelial RWPE-1 cells were incubated up to 16 h in the culture medium containing apigenin. At each time point the cells were harvested and extracted, and apigenin levels were measured. The time courses of apigenin uptake by RWPE-1, LNCaP, PC-3 and DU145 cells are shown in **figure 2A**. The initial uptake of apigenin was rapid up to 2 h, followed by a slower but sustained uptake that reached a plateau after 16 h post-incubation. Furthermore, incubation of 20 µM apigenin for 16 h exhibited an accumulation of 1.48 nmoles/million cells which was higher than in PC-3 and DU145 cells by a factor of 1.89 and 2.71, respectively. Almost similar uptake of apigenin was noted in androgen-responsive LNCaP cells as in RWPE-1 cells **(** **Figure 2B** **)**. These results indicate a preferential uptake of apigenin by RWPE-1 and LNCaP cells was higher compared to PC-3 and DU145 cells.

### Uptake Kinetics of Apigenin in Human Prostate Cancer LNCaP Cells

**Figure 3:**
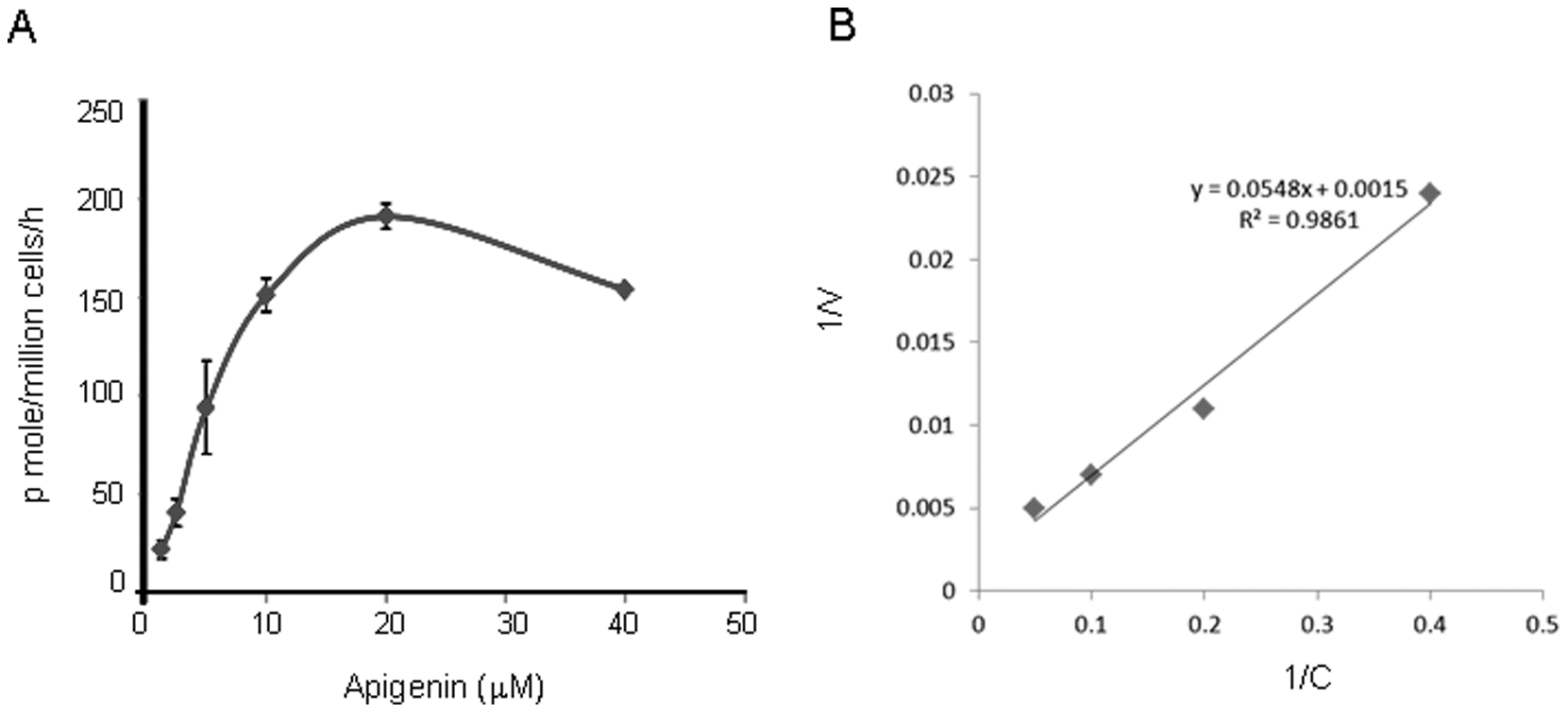
Kinetics of apigenin uptake by human prostate cancer LNCaP cells. (**A**) Dose-dependent kinetics of apigenin uptake in LNCaP cells incubated for 6 h with apigenin at concentration ranging from 1.25 µM to 40 µM. The rate of apigenin uptake was measured as (cellular apigenin) million cells^−1 ^h^−1^. Points±SD of experiments performed three times. (**B**) Data evaluated using Michaelis-Menten kinetics by constructing a reciprocal plot between 1/C and 1/V. Details are described in materials and methods section.

Because of higher uptake of apigenin by LNCaP cells, these cells were further studied for absorption kinetics and the results are shown in **figure 3**. The 6 h time point was selected because it is before the plateau level and within the linear range of apigenin intake in the previous experiment. The kinetics of apigenin uptake by LNCaP cells was saturable and concentration dependent. Apigenin absorption kinetics showed a K_m_ value of approximately 5 µmole/litre and a V_max_ of 190 pmoles/h/million cells **(** **Figure 3A** **)**. A reciprocal plot between hours of incubation and absorption rate is linear which showed proportionality between these parameters (R^2^ = 0.9861) **(** **Figure 3B** **)**.

### Sub-cellular Distribution of Apigenin in LNCaP Cells

**Figure 4:**
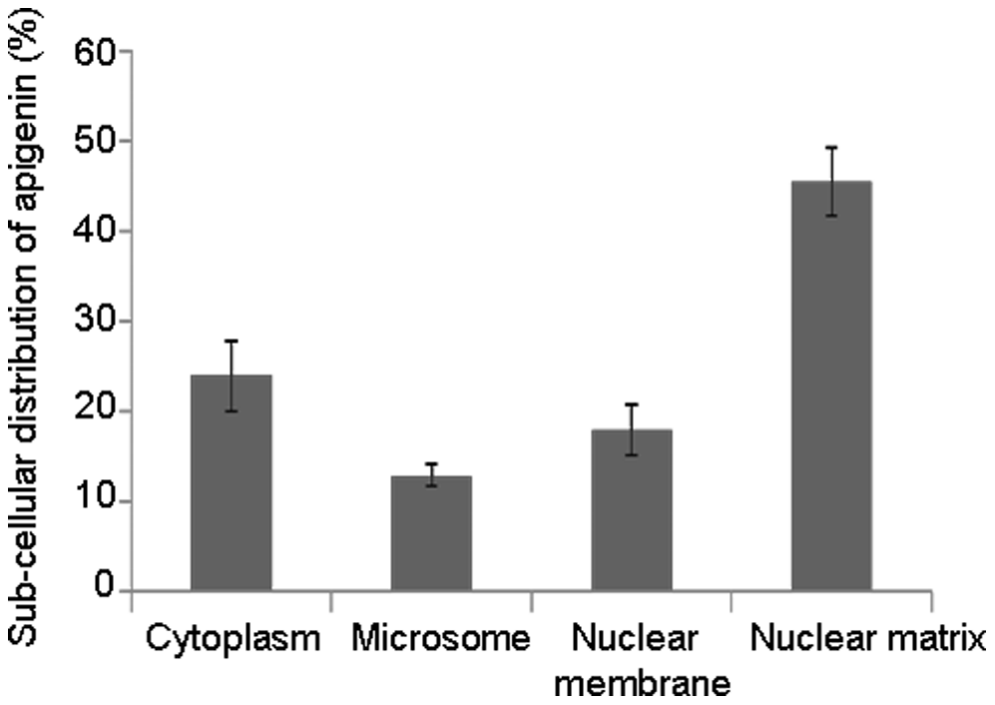
Sub-cellular distribution of apigenin in human prostate cancer LNCaP cells. The cells were incubated with 20 µM apigenin for 48 h with approximately 5×10^6^ cells and processed for different fractions. Bars±SD of experiments performed three times. Distribution is represented as 100% apigenin in all the fractions. Details are described in materials and methods section.

Next we determined intracellular localization of apigenin within LNCaP cells. The cells were separated into four sub-cellular fractionations by lysis in hypotonic buffer and then differential centrifugation followed by quantitative measurement of apigenin by HPLC. As shown in **figure 4**, sub-cellular fractionation results exhibit that nuclear matrix retains the highest concentration of apigenin (45.3%), followed by cytosol (23.9%), nuclear membrane (17.9%) and microsomal fraction (12.9%), respectively. This preferential accumulation of apigenin in the nuclear matrix suggests its interaction with the nucleic acids.

### Interaction of Apigenin with Calf Thymus DNA

**Figure 5:**
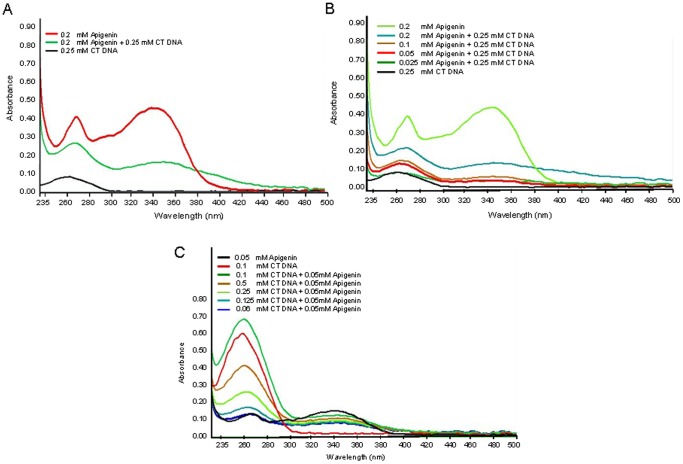
Interaction of apigenin with calf thymus (CT) DNA. (**A**) UV-Vis spectra of CT-DNA, apigenin and apigenin+CT-DNA. (**B**) UV-Vis spectra of CT-DNA with varying concentration of apigenin ranging from 0.025 mM to 0.2 mM in solution. (**C**) UV-Vis spectra with 0.05 mM apigenin along with varying concentration of CT-DNA ranging from 0.06 mM to 0.1 mM in solution. Absorption spectra of solution were recorded from 230 nm to 500 nm using Nanodrop. The experiment was repeated three times with similar results. Details are described in materials and methods section.

In the next experiment, we determined the interaction of apigenin with calf thymus (CT) DNA. As shown in **figure 5A**, the absorption spectra of solution containing apigenin, CT-DNA and apigenin+CT-DNA were recorded from 230 nm to 500 nm. The absorbance value of DNA increased at 260 nm upon addition of apigenin and CT-DNA in accordance with the Beer’s Law **(** **Figure 5B & C** **)**. This indicates that apigenin might have intercalated between the strands of DNA thereby increasing the absorption of DNA due to the unwinding of DNA double helical structure, which has been previously reported with other plant flavonoids [28]–[30].

### Protection of Oxidative DNA Damage by Apigenin

**Figure 6:**
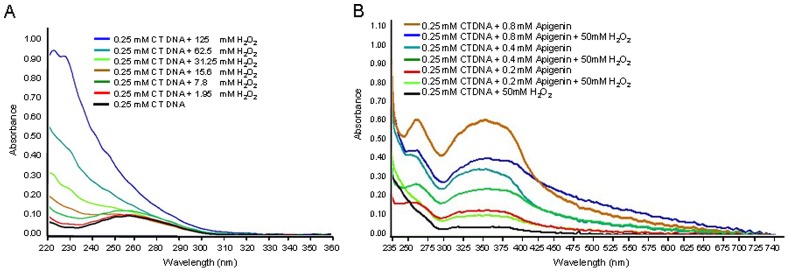
UV-Vis spectra of H_2_O_2_ and its quenching by apigenin. (**A**) UV-Vis spectra of calf thymus (CT) DNA incubated with varying concentration of H_2_O_2_ ranging from 1.95 mM to 125 mM. (**B**) Quenching of UV-Vis spectra by apigenin after H_2_O_2_ treatment. CT-DNA was incubated with varying concentration of apigenin ranging from 0.2 mM to 0.8 mM followed by H_2_O_2_ treatment. The experiment was repeated three times with similar results. Details are described in materials and methods section.

To determine the antioxidant potential of apigenin, CT-DNA was incubated with increasing concentration of H_2_O_2_. As shown in **figure 6A**, incubation with H_2_O_2_ increased the peak absorbance in a dose-dependent manner. However, the presence of apigenin from 0.2 mM to 0.8 mM prevented H_2_O_2_-mediated damage to DNA as shown by the restoration of the peak absorbance of DNA **(** **Figure 6B** **)**.

### Reduction of Oxidative Stress and H_2_O_2_-mediated Oxidative DNA Damage by Apigenin

**Figure 7:**
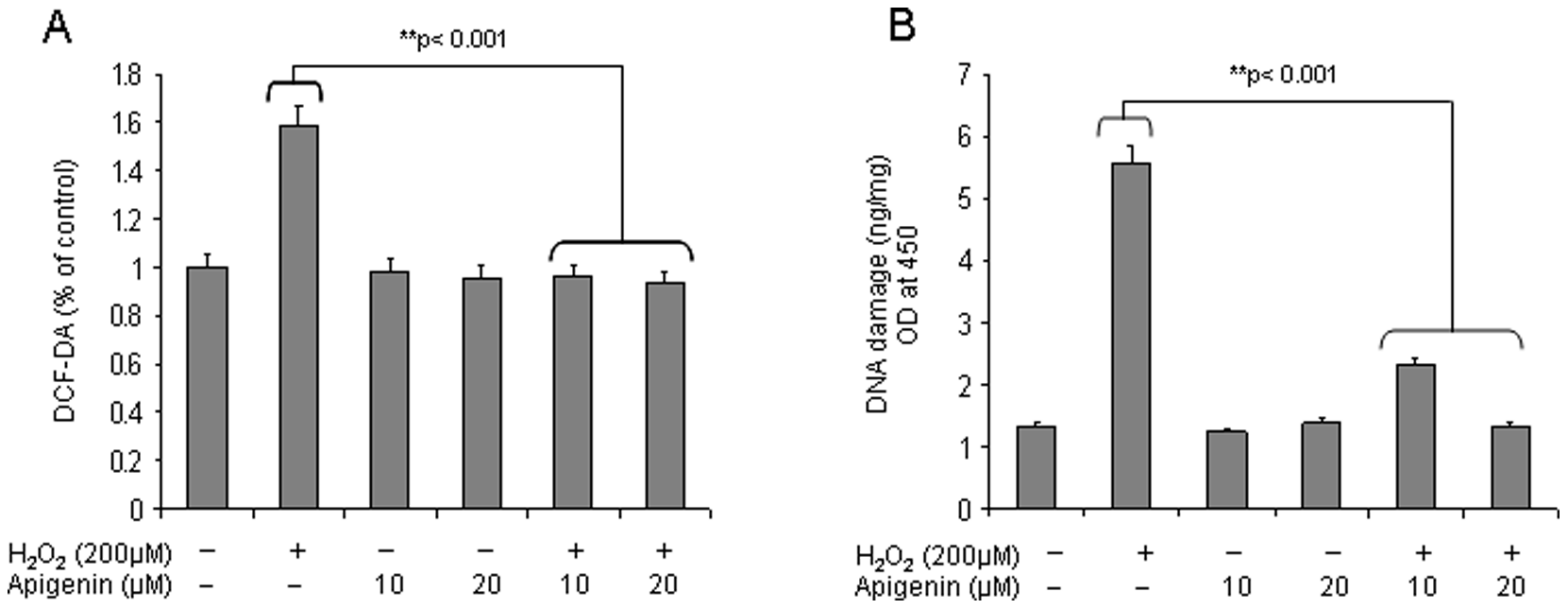
Effect of apigenin on reactive oxygen species (ROS) generation and 8-hydroxy-2′-deoxyguanosine (8-OHdG) levels with H_2_O_2_ in transformed human prostate epithelial RWPE-1 cells. (**A**) ROS assay with DCF-DA on RWPE-1 cells treated with 10 µM and 20 µM apigenin for 16 h followed by 200 µM H_2_O_2_ incubation for 6 h. (**B**) 8-OHdG levels in RWPE-1 cells treated with 10 µM and 20 µM apigenin for 16 h followed by 200 µM H_2_O_2_ incubation for 6 h. Bars±SD of experiments performed three times. **P<0.001, compared to H_2_O_2_ treated group. Details are described in materials and methods section.

Next we determined the protective effect of apigenin from oxidative stress. Exposure of RWPE-1 cells with H_2_O_2_ caused a significant increase in reactive oxygen species (ROS) generation as measured by the addition of DCF-DA in the culture medium, which converts to highly fluorescent dichlorofluorescein in the presence of intracellular ROS. Pretreatment of cells with 10 µM and 20 µM apigenin caused significant decrease in ROS generation (p<0.001), compared to H_2_O_2_-treated cells **(** **Figure 7A** **)**. We also determined the levels of 8-OHdG, a hallmark of oxidative stress DNA base damage. As shown in **figure 7B**, the levels of 8-OHdG in DNA were significantly higher in H_2_O_2_ -treated cells than in untreated cells or in cells treated with apigenin. Apigenin significantly decrease the levels of 8-OHdG induced by H_2_O_2_ treatment (p<0.001). These results suggest that apigenin has the ability to protect the cells from oxidative-mediated cellular injury.

### Protection of Human Prostate Epithelial Cells from H_2_O_2_-induced Cell Death by Apigenin

**Figure 8:**
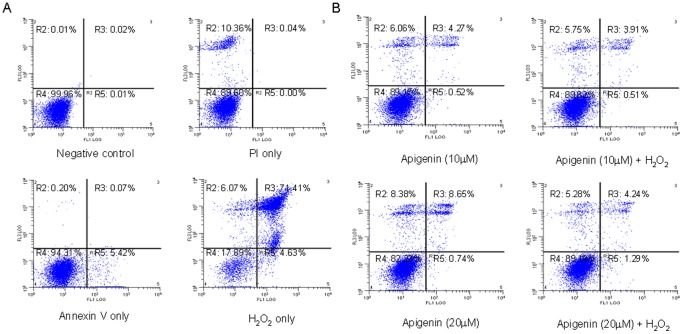
Effect of apigenin on H_2_O_2_ -mediated RWPE-1 cell death. (**A**) Effect of H_2_O_2_ on cell death in RWPE-1 cells. The cells were treated with 200 µM H_2_O_2_ for 6 h. Negative, PI and annexin V only treatments are included as controls. (**B**) Cells were treated with 10 µM and 20 µM concentration of apigenin for 16 h followed by 200 µM H_2_O_2_ incubation for 6 h, stained with PI and annexin V for 15 min and analyzed using fluorescence activated cell sorter (FACS). Data shown is representation of FACS graphs analyzed two times in duplicate. Details are described in materials and methods section.

Next we examined whether apigenin could decrease H_2_O_2_ -mediated cellular injury and death of RWPE-1 cells. The cells were treated with 200 µM H_2_O_2_ for 6 h. As shown in **figure 8A**, exposure of cells to H_2_O_2_ resulted in 71.4% increase in annexin V staining demonstrating increase oxidative stress-mediated apoptosis. To confirm the protective effect of apigenin, the cells were treated with 10 µM and 20 µM apigenin for 16 h and later exposed to 200 µM H_2_O_2_ for 6 h. Treatment of RWPE-1 cells with apigenin resulted in a marked decrease in H_2_O_2_-mediated apoptotic cell death **(** **Figure 8B** **)**. Treatment with apigenin alone did not induce substantial apoptosis in these cells. Overall, these results suggest that apigenin has the ability to protect prostate epithelial cells from H_2_O_2_-mediated cellular injury and apoptosis.

## Discussion

In this study we explored cellular uptake of apigenin in transformed human prostate epithelial cells and various prostate cancer cells. We evaluated its sub-cellular distribution, DNA binding activity, and quenching of H_2_O_2_-induced ROS and oxidative stress in *in vivo* cell cultures and *in vitro* systems, using calf thymus DNA. Our results, for the first time, demonstrate that apigenin preferentially accumulates in the nuclear matrix, binds with the DNA to reduce oxidative DNA damage and apoptosis in prostate epithelial cells.

Reported studies to date indicate that frequent consumption of plant-based food products rich in flavones may be beneficial in reducing the risk of prostate cancer [31]. Apigenin, a plant flavone, has received considerable attention due to its wide distribution in the plant kingdom and because of its health benefits and chemopreventive properties [16 and references therein]. Many studies have demonstrated that apigenin possesses a wide range of biological activities, including anticancer, antiviral, antibacterial, antioxidant and anti-inflammatory effects [16]–[20]. These biological activities are considered to be related to its intracellular distribution and interaction with several biological targets. In our studies, apigenin accumulation in human prostate cancer cells is in the following order of magnitude: LNCaP>PC-3>DU145 cells. The highest level of apigenin accumulation occurs in transformed human prostate epithelial RWPE-1 cells. These results indicate that apigenin preferentially accumulates in cells which possess functional androgen receptor (AR). Furthermore, studies demonstrate that androgens, via the androgen receptor, induce oxidative stress in normal and prostate cancer cells [32], [33]. Androgens modulate the production of ROS via both the induction of fatty acid oxidation in the mitochondria and via the induction of NADPH oxidase activity [34]. Our previous studies demonstrate that high caloric intake increases oxidative stress in the mouse prostate via the NOX family of ROS-generating NADPH oxidases and sustained activation of NF-КB and STAT-3 transcription factors [35], [36]. In the present study, apigenin accumulation in cells with functional AR may have the potential to interfere with AR signaling. Previous studies have demonstrated that apigenin interferes with AR signaling and inhibits androgen-responsive genes [37]. However, further studies are needed to clarify the interactions between AR and apigenin.

The absorption and bioavailability of flavonoids remain critical issue in evaluating its cancer preventive effects. We determined how cellular uptake of apigenin changes in human prostate cancer LNCaP cells by exposing them to various concentration of apigenin up to 40 µM. The Michaels-Menton kinetics of cellular uptake was characterized by saturation at high apigenin concentration suggesting that the process of apigenin uptake by LNCaP cells might be through passive diffusion. The K_m_ value for the uptake of apigenin is high, relative to the concentration obtained in human and mouse plasma, which indicates that apigenin has low binding affinity with plasma proteins. Thus far, there are no reports of apigenin receptors or binding proteins that facilitate its cellular uptake.

Growing evidence suggests that chronic inflammation with low levels of reactive oxygen species (ROS) production plays an important role in causing DNA damage and development of cancer [8], [9]. Reactive oxygen species oxidize DNA bases, leading to mutation and DNA hypermethylation [10], [11]. Reactive oxygen species induce peroxide formation in membrane lipid molecules, altering the physiochemical properties of membranes and damage membrane-bound proteins and other macromolecules. In addition, reactive oxygen species exert deleterious chemical effects on proteins that can alter normal cellular functions. Our previous findings in a prospective 5-year follow-up study in needle biopsy specimens demonstrate a strong association between chronic prostatic inflammation, premalignant, and malignant changes in the prostatic epithelium [9]. Mechanistically, inflammatory cells are drawn to the site of inflammation and consequently myeloperoxidase and phagocytic NADPH oxidase derived ROS are released. Parallel secretion of inflammatory cytokines acerbates inflammatory process via NF-КB and STAT-3 activation and favors cellular ROS formation [38]. These pro-oxidative changes in the prostate microenvironment in combination with genetic susceptibility such as defects in encoding for GSTP1 and DNA repair enzymes may contribute to initiation of prostate carcinogenesis [39]. In the present study we demonstrate that apigenin exposure significantly quenches ROS generation and protects prostate epithelial cells from oxidative DNA damage, and may thereby inhibit carcinogenesis. Furthermore, we have shown that apigenin suppresses NF-КB activation. Additional studies are needed to precisely evaluate the effects of apigenin in decreasing inflammatory mediators and epigenetic modification.

Oxidative stress initiates DNA modification and mutagenic lesions that contribute to pathologic processes in various diseases, including cancer [40]–[42]. 8-hydroxy-2′-deoxyguanosine (8-OHdG) is a major base product that is formed after an oxidative insult to DNA [43]. Large amounts of 8-OHdG are produced in mammalian cells, either as a by-product of normal oxidative metabolism or as a result of exogenous sources of ROS. Studies have shown that 42% of men aged 55–80 years exhibit prostatic DNA damage, reflected by levels of 8-OHdG, which results from oxidative modification of guanine [2], [44]. Oxidative damage to the DNA base leads to a point mutation by an A→T substitution when incorporated into DNA [45]. It has been demonstrated that hydroxyl radical (OH**^.^**), singlet oxygen (O_2_^−^), or peroxinitrite anion (ONOO^−^) is responsible for the formation of 8-OHdG. Levels of 8-OHdG in tissues may increase either because there is a strong DNA damaging stimulus or because one of the specific DNA repair mechanism is deficient [4]–[8]. Our studies demonstrate that apigenin protects against oxidative DNA damage. Further studies are needed to clarify the mechanistic pathways responsible for this effect.

There is now considerable published scientific data regarding the interaction of plant flavones with various proteins and lipids [28]–[30], [46], [47]. Our work on spectroscopic study of the interaction of apigenin with calf-thymus DNA *in vitro* suggests that classic intercalation is the dominant binding mode and may affect reactions associated with enzymes on DNA molecules. In particular, apigenin has been shown to inhibit the activities of various proteins attached to DNA, such as DNA polymerase, cAMP-response element binding proteins, DNA topoisomerase, and chromatin modifying enzymes, including histone deacetylases [48]–[51]. Ours is the first study demonstrating the sub-cellular distribution of apigenin, documenting its interactions with DNA and elucidating its role in inhibiting oxidative stress within cells. Although several mechanisms by which apigenin might prevent prostate cancer have been demonstrated and/or are under investigation, our data are consistent with the concept that its antioxidant activity in the nucleus accounts for its documented capacity to serve in the chemoprevention of prostate cancer.
